# Book Reviews

**Published:** 1803-10-01

**Authors:** 


### CRITICAL ANALYSIS

OF THE

### RECENT PUBLICATIONS

ON THE

### DIFFERENT BRANCHES OF PHYSIC, SURGERY, AND MEDICAL PHILOSOPHY.

---

*A Concise History of the Autumnal Fever which prevailed in the Borough of Wilmington, in North America, in the Year* 1802. *By* Dr. JOHN VAUGHAN. 8vo. pp. 32. Wilmington, (Del.)

A manuscript account of this distemper at Wilmington was sent, as we have understood, to Philadelphia for publication. The author was led to believe it would have been inserted entire in a collection of Essays then preparing for the press. But he was much disappointed to find that "a mutilated abstract only was published." After having asked some explanation from his correspondents concerning this manner of treating his performance, and obtaining but little satisfaction, he resolved to do himself justice by printing the whole under his own eye.

Though this is a short, it is an intelligent account of a disease which afflicted the place in which the writer resides, in the fall of

1802. It is a valuable piece of local medical history, drawn up by a physician who describes what he saw, and who lived through the scenes of calamity and terror which he witnessed. Dr. Vaughan has, however, not confined himself to a mere delineation of symptoms, and a recital of prescriptions; he investigates the origin and progress of the malady, and endeavours to trace it to some external or internal source. He declares that, with all his vigilance, he cannot discover it to have been brought from any place whatever, either by water or by land, by persons or by goods. He examines, with care, the allegations against a young woman, and a schooner, charged with having *imported contagion* into Wilmington, the former from Philadelphia, and the latter from Port Republican; and finds them quite unfounded in reality, no better supported than similar tales usually are. But we shall give Dr. V.'s own words. (p. 15.)

"On a review of the preceding narrative, it appears that the disease took rise in the narrow part of King Street and the adjacent district, progressing, with irregular steps, over the lower parts of the town, and finally encroaching on the district north of Third Street; but was principally confined to the district south-east of Market and Third Streets.

"The more prominent facts relating to the origin of the disease stand thus:—1. Ann Davidson, the only person who was or could be suspected of introducing contagion among us, came from Philadelphia in the beginning of August, to her father's house in King Street, and was reported to be affected with contagious fever on the 15th. She recovered, without suffering any of the more violent symptoms of malignant disease, and removed into the country.

"2. The family in which she resided consisted of ten persons, all of whom remained well until the 7th of September, when her mother had an attack of fever, after a journey of ten miles into the country, performed partly on foot, and partly in an open cart.

"3. In the mean time, T. Musgrove's son, Ann Hadley, Capt. West's girl, Mr. Cloud, and Hannah Robinson, in the same square; and R. Hagin's son, J. Warner's girl, E. Dale, and others, in different directions, were attacked with malignant fever.

"4. If Ann Davidson's disease were contagious, and the only source of future disease, is it not reasonable to suppose that some one or more of the ten persons, confined in a small house with her, would have been the first affected by the contagion? The reverse was the fact.

"5. The first serious alarm of malignant fever took place in the first week of September. The cases were principally, but not altogether, confined to King Street. The square between Second and Front Streets, whose filthy cellars *was* a matter of notoriety, and a subject of common complaint, was the more concentrated seat of disease.

"6. The cases specified evince a correspondency of dates, indicating the action of a common cause, and precluding the more slow and successive routine of human contagion, from one diseased person to another.

"7. The

" 7. The disease was suspended by the great change of weather which took place on the 5th of September, and resumed a formidable shape about the 9th, and became general in the southern district against the 25th of the month.

" 8. When the disease became alarming a second time, I personally inquired into the preliminary circumstances of every case that occurred, for the purpose of tracing their origin; and none of them could be reasonably imputed to contagion from the previously sick.

" 9. The disease was evidently subservient to the states of the weather, in declining in frequency and force in warm clear weather, and re-assuming a formidable shape on every change to coolness and moisture of the atmosphere.

" On the most liberal view which can be taken of the rise and progress of the disease, with an unrestrained examination of the facts, as they occurred, I see no reason to suppose the insidious malady was of foreign origin, or specifically contagious. On the contrary, I am firmly impressed with the belief, that it was the endemic fever of Autumn aggravated to a pestilential grade, by local filth, and the tropical state of the season, in conjunction with an epidemical state of atmosphere, which appears to have influenced the diseases of our country since the memorable year 1793. This belief is further predicated on the following facts and inferences:

" 1. An epidemical state of atmosphere, favouring the occurrence of malignant fever, was evinced by the usual premonitary forms of disease.

" The measles were epidemic in the fall of 1801, and declined during the winter, giving place to the scarlet fever. The last winter was unusually mild, which gave birth to the swellings of the glands, croup, and the influenza, in April and May, 1802. June was, as usual, comparatively healthy, but chequered with some cases of cutaneous disease. In July the putrid sore throat occasioned considerable alarm; and, during the latter part of July and early part of August, the eruptive state of fever was extremely afflicting to children, and seemed to supercede the ordinary appearance of cholera infantum. In some cases general ulceration of the glands of the neck and axillæ were so obstinate as to require a complete course of alterative remedies.

" 2. The season became tropical in the middle of August. The weather, from being uncommonly cool, suddenly became extremely hot, varying from 80 to 90 degrees, with frequent gusts of rain and lightening. In the evenings of the 29th, 30th, and 31st, there were excessively violent thunder-storms from the westward, with torrents of rain. It was remarkable, during the autumn, that all our sudden and violent rains came from the westward, and commonly extended but a few miles along the Delaware. In misty weather the wind was mostly north-east. But the winds were unusually variable, not unfrequently traversing the compass in twentytwenty-four hours. The changes of the weather were proportionally sudden.

"3. Myriads of mosquitoes infested the lower parts of the town from July until frost, having gradually diffused themselves over the borough in September. The eldest of our inhabitants do not recollect this insect being troublesome here in any previous season; while the unanimous report of persons from the fenny counties of Kent and Sussex, the annual haunt of these winged pests, was, that they were unusually free from them.

"4. The sources of noxious effluvia in the southern and flat part of the town were much increased by a regulation, but partially executed, for bringing the streets to an uniform descent from the summit of the hill. A number of cellars were filled with water; a new dock formed; and the gutters lowered in some places and raised in others, forming numerous depositories of filth. These circumstances, added to the nuisance in King Street, rendered the air of that quarter offensive to the smell in the day-time, and doubly so at night. After the 15th of September the air had a taint resembling bilge-water, especially after a light shower of rain and in the night, and more sensibly recognized by persons coming immediately from a higher region.

"5. The fogs which collected in the evenings were suspended on the flats during the nights, gradually becoming more compact in the mornings, and mostly passed off in a dense cloud towards the Delaware, between seven and ten o'clock. This semi-circuit of the fogs, from Market Street southward and eastward, was the seat of concentrated disease. Those fogs were condensed miasmata of fever in a familiar garb.

"The seat of disease was so well defined until the 15th of October, that the inhabitants north of Third Street felt but little apprehension; and, as the fogs became diffused, a few scattering cases of disease appeared, and removal was the only mean of safety.

"6. The poisonous matter exciting disease was evidently a constituent part of the fogs. Many persons visited the infected district in clear weather, and in the day-time, without injury, and several of the same persons contracted disease by a single exposure in the night-time, after the fog had collected. It also is remarkable, that the disease generally attacked in the night-time.

"7. The non-contagious nature of the disease was repeatedly attested by persons sickening after removal from the lower to the higher parts of the town, and being nursed with every attention, and dying without communicating the malady to their attendants. Also, two sailors had the disease, on board of different vessels, at separate wharfs, without affecting their companions.

"It is not denied that the more malignant cases of disease may be incidentally contagious, or rather re-infectious, under circumstances favouring a new chemical combination of the venomous offsprings of filth and putrefaction.

"8. A noxious state of atmosphere was manifested by the lingering ering state of convalescents who remained in the contaminated region, while those who removed into the country were speedily restored to health.

"9. The indigenous nature of the disease was evidently characterised by the ultimate sameness of every form and grade of fever. After the middle of September, the subordinate forms and grades of fever, not arrested within forty-eight or seventy-two hours, invariably passed on to the malignant grade of disease. No matter how slight the attack, nor who the subject, the livery of pestilence sooner or later appeared: and valetudinarians, cases of pulmonary consumption excepted, suffered in the common fatality.

"Lastly. The rise, progress, and confined state of the disease; the manner in which the fluctuating malady corresponded with the varying states of the weather; the non-communication of disease to the attendants on the sick, when out of the original sphere of infection; and the sporadic appearance of disease in other parts, after the more extended fog on the 15th of October; with the final termination of the progress of infection by a single frost—are, in my opinion, evidences striking as the nature of the case will possibly admit, that the multiform disease which afflicted us was not of foreign origin, not specifically contagious.

"I believe that a proper attention to the internal condition of this borough, and preserving the adjacent marshes in their present state of cultivation and improvement, by numerous drains, would be the surest means, under Providence, of securing us against a repetition of the calamity."

We think Dr. V. is entitled to much credit for the impartiality and intrepidity he has shown, and we hope his conduct will be an example to other gentlemen in the profession to observe, and relate what they shall see, with equal fidelity. We shall thus, by degrees, correct a considerable part of the errors which overhang the history of febrile distempers.

---

*Practical Observations on Surgery, illustrated with Cases, by* WILLIAM HEY, *Esq. F. R. S. Member of the Royal College of Surgeons, in London; Honorary Member of the Royal Medical Society of Edinburgh, and of the Literary and Philosophical Society of Manchester; and Senior Surgeon of the General Infirmary at Leeds.* London, 1803. pp. 537, 8vo.

THESE valuable observations are of a kind the best calculated of all others to render a real and essential service to the art which they illustrate. They contain some of the most important remarks on various parts of surgery, made by a practitioner of great and deserved eminence, who for many years has filled a leading station in his profession. Little scope is here given to conjecture, nor is any observation introduced, which has not the basis of personal experience, and both the character of the author and the internal evidence which appears on the face of the work, give it the stamp of perfect authenticity.

The contents of this publication are a number of detached Essays on miscellaneous subjects of Surgery, given without arrangement or connexion with each other, and extracted almost entirely (as the author informs us in the preface) from his case-book, which he has been in the habit of keeping ever since his commencement of practice; a rich repository of excellent surgery to judge by the example before us! We shall take some notice of each Essay.

The first contains remarks on fractures of the skull. Mr. H. begins by objecting to a point of practice laid down by the celebrated Pott, in his excellent Treatise on Injuries of the Head, which is, that previous to the operation of trepanning, a portion of the scalp large enough to allow of the examination of the fracture, and the free employment of the trephine, should be actually removed by the knife. To this Mr. Hey objects with propriety, as unnecessary and injurious, inasmuch as all the advantage to be derived from a free examination of parts, may be gained by a large crucial incision into the scalp, and the natural integuments still remain as a covering to the bone and dura mater, after the operation. Indeed this method is so consonant to general experience, that we believe it is now almost universally adopted, and we may with reason be surprized at this maxim of one of our most excellent surgical writers, since, in other parts of the same essay, he is very explicit in directions for saving the scalp, when injured, if there is the least chance of its being brought to re-unite to the skull. A very important improvement in the instrument for removing the necessary portion of the cranium, is next given by our author. It is to substitute a flat, straight, or flat convex saw to the circular instrument in common use. In truth, it must be acknowledged to shew a great want of contrivance to employ only a single method of working on the cranium to be adapted to all accidents of this bone; and, considering this operation in a workman-like manner, we think that much benefit may be derived from the proposed improvement; we shall give the author's words:

"When a broken fragment of bone is driven beneath the sound contiguous part of the cranium, it frequently happens, that the extraction cannot be executed without removing some of the unbroken part, under which the fragment is depressed. This might generally be effected with very little loss of sound bone, if a narrow portion of that which lies over the broken fragment could be removed. But such a portion cannot be removed by the trephine. This instrument can only saw out a circular piece. And as, in executing this, the central pin of the saw must be placed upon the uninjured bone, it is evident, that a portion of the sound bone, greater than half the area of the trephine, must be removed at every operation. When the broken and depressed fragment is large, a repeated application of the trephine is often necessary, and a great destruction of sound bone must be the consequence.

"When the injury consists merely of a fissure with depression, a small enlargement of the fissure would enable the surgeon to introduce the point of the elevator, so as to raise the depressed bone. But a small enlargement of the fissure cannot be made with the trephine. When it is necessary to apply the elevator to different parts of the depressed bone, a great deal of the sound cranium must be removed, where a very narrow aperture would have been sufficient.

"The same reasoning will apply to the case of openings made for the purpose of giving a discharge to extravasated blood, or matter.

"If a saw could be contrived, which might be worked with safety in a straight, or gently curvilineal direction, it would be a great acquisition to the practical surgeon. Such a saw I can now with confidence recommend, after a trial of twenty years, during which time I have rarely used the trephine in fractures of the skull."

Some very satisfactory cases are added, to shew the superior advantage of this instrument to the circular saw, which applies more peculiarly to those instances of fracture with great depression, where the injured bone is not loose enough to be removed by the forceps, and must therefore be got at by means of perforating the sound contiguous part of the skull. Two cases are also given, in which this saw was used with advantage in a necrosis of the tibia to separate the diseased portions of bone.

The cataract of the eye is the subject of the next Essay, on which the author has evidently bestowed much attention. The leading object is to recommend the operation of couching in preference to extraction, though indeed the author's experience hardly allows him to draw the *comparison* between the two methods, as it has been almost entirely confined to the couching needle. The instrument employed by Mr. H. differs in some material points from the needle commonly in use, being much shorter, smaller, and not spear pointed, but more resembling a common awl. The following is the exact description of it: "The length of the needle is somewhat less than an inch. It would be sufficiently long if it did not exceed seven-eighths of an inch. It is round, except near the point, where it is made flat by grinding two opposite sides. The flat part is ground gradually thinner to the extremity of the needle, which is semicircular, and ought to be made as sharp as a lancet. The flat part extends in length about an eighth of an inch, and its sides are parallel. From the place where the needle ceases to be flat, its diameter gradually increases towards the handle. The flat part is one-fortieth of an inch in diameter. The part which is nearest the handle is one-twentieth of an inch. The handle, which is three inches and a half in length, is made of light wood stained black. It is octagonal, and has a little ivory inlaid in the two sides, which correspond with the edges of the needle."

The advantages which the author describes as resulting from this

 form form of the instrument, are, first, that its length is so nearly adapted to the dimensions of the natural eye, that the surgeon can better judge of the direction and extent of the wound when he is unable to see the point. Secondly, that as the needle becomes gradually thicker towards the handle, it will remain fixed to that point of the sclerotis to which it has pushed it, and will give the eye more steadiness during the depression, and will also prevent the escape of any part of the vitreous humour. Thirdly, that as it has no projecting edges, like the spear-shaped needle, it will be less liable to wound the iris or ciliary processes; and fourthly, that having no projecting point, it will not readily injure the edges of the pupil during the motion of depression, which the common needle is apt to do.

The description of the operation with this instrument is clearly expressed, and very precise. It does not differ materially from that of other operators. Mr. H is in the constant habit of couching the right eye with the left hand, and to acquire this ambidexterity, he gives the useful advice to the surgeon to accustom himself to bleed with the left hand.

The testimony of our author to the excellence of this operation is indeed strong and irrefragable, and certainly ought to have due weight against the opposite testimony of some very eminent oculists who uniformly prefer extraction. The peculiar characteristic of Mr. Hey's practice in this operation, (besides the form of the instrument) is that of caution in not attempting too much at first, and he does not hesitate to leave a great deal for future operations, rather than endanger the safety of the organ by violent inflammation. Thus we find, in the cases which are subjoined, the operation repeated as often as twelve times, and gaining ground by slow degrees in removing the opacity, attended finally with perfect success. It is owing to this caution also, that Mr. H. has been enabled to operate successfully even in partial adhesions of the iris, and in other cases of difficulty, which in general have been thought to forbid the use of the needle. Baron Wenzel, in his valuable treatise on this disease, mentions as an objection to couching, that "the pain produced by the puncture of the retina and the ciliary nerves, is often followed by a suppuration of the eye." Our author's answer to this objection is worthy of remark.

"I have now practised the operation of couching pretty frequently for thirty-three years, though I have not kept a list of all the patients upon whom I have operated. I have also seen the operation performed frequently by my colleagues at the Leeds Infirmary; but never yet saw an instance of a suppuration of the eye, in any patient who has come under my care in private practice, nor in any case that has occurred at our public hospital."

Now, if in the course of a practice so extensive as Mr. Hey's appears to have been, and with very frequent repetitions of the operation on the same subject, no such mischief as Baron Wenzel threatens threatens has ever occurred, we must allow this hazard to be much over-rated.

Little is added to the pathology of the disease in this Essay, except to shew from actual experience the fallacy of several of the diagnostics of hard and soft cataract, curable and incurable, which several authors have attempted to establish.

In the third Essay the disease of strangulated hernia is considered. The first treatment of this disease does not much differ from that in common practice. Bleeding is advised only in very full plethoric habits, purgative medicines always to be avoided, opiates used very freely, and especially tobacco clysters. The experience of sound surgery will, we apprehend, agree with our author in advising an early operation. We shall give his words:

"When I first entered upon the profession of surgery, in the year 1759, the operation for the strangulated hernia had not been performed by any of the surgeons in Leeds. My seniors in the profession were very kind in affording me their assistance, or calling me into consultation when such cases occurred; but we considered the operation as the last resource, and as improper until the danger appeared imminent. By this dilatory mode of practice I lost three patients in five upon whom the operation was performed. Having more experience of the urgency of the disease, I made it my custom, when called to a patient who had laboured two or three days under the disease, to wait only about two hours, that I might try the effect of bleeding (if this evacuation was not forbidden by some peculiar circumstances of the case) and the tobacco clyster. In this mode of practice I lost about two patients in nine upon whom I operated. This comparison is drawn from cases nearly similar, leaving out of the account those cases in which a gangrene of the intestine had taken place.

"I have now, at the time of writing this, performed the operation thirty-five times; and have often had occasion to lament that I had performed it too late, but never that I had performed it too soon. There are some cases so urgent, that it is not adviseable to lose any time in the trial of means to produce a reduction. The delay of a few hours may cut off all hope of success, when a speedy operation might have saved the life of the patient."

The part which causes the strangulation in femoral hernia, Mr. Hey asserts, is not Poupart's ligament, but another ligament, to which he gives the name of *femoral*, running nearly in the same direction as Poupart's, but rather in an ascending direction, and situated below and behind it. A plate of this ligament is given. Here the author also refers to the treatise of Gimbernat, who has described these parts with great accuracy.

Many cases are added to this very important chapter, and the author, in particular, insists upon the care to be taken in disposing of the omentum in the operation for hernia, and the hazard

 incurred incurred by hæmorrhage of this substance, when returned into the abdomen with the vessels not properly secured.

An account of two uncommon varieties of hernia, one of the congenital scrotal kind, and the other of the umbilical, conclude this subject. *(To be continued.)*

---

*Experiments and Observations on the Cortex Salicis Latifoliæ, or Broad-leafed Willow Bark; illustrated by a coloured Plate, interspersed with general Observations and Remarks on the different Species of the Cinchona, &c. General History and progressive Introduction of the Salix Latifolia; with a Variety of Experiments, tending to elucidate its Properties. Illustrated by Cases demonstrating its superior Efficacy above the Cinchona in various Diseases, more particularly that Branch of the Healing Art, termed Medical Surgery*; *by* G. WILKINSON, Member of the College of Surgeons, London, &c. Newcastle upon Tyne. 8vo. pp. 118. 1803.

THE virtues of the various species of willow bark have long been ascertained and fully established, but the salix has not hitherto been formally received into our materia medica. In 1792, Mr. James, of Hoddesdon, in Hertfordshire, published a pamphlet recommending the salix latifolia (caprea) in strong terms, as a substitute to the cinchona; and in 1798, Mr. White, apothecary to the Bath Infirmary, added a valuable testimony to the efficacy of this bark. The author of the treatise before us has done the same, and has attempted to shew, by chemical experiments, the ground of its excellency.

The sensible properties of this bark are, in smell, scarcely perceptible; in taste, considerable astringency with a slight degree of bitterness, which last however goes off almost entirely on drying. It is in the *astringency alone* that the medicinal virtue of this bark seems to reside, and the whole object of the author's experiments is directed to this point. Indeed, his whimsical motto from the grave-digger's scene in Hamlet, is an indication to the reader what to expect. "A tanner will last you nine years." "Why he more than another?" "Why, sir, his hide is so tanned with his trade, that he will keep out water a great while."

The author gives a number of comparative experiments on the quantity of tannin contained in the following vegetables; the tormentil root, oak, cinchona of each species, angustura, and the willow bark in question. He determines their antiseptic property by their effect on animal flesh in retarding putrefaction; their tanning power, by immersing pieces of skin in the different solutions; and the actual quantity of tannin by the very accurate method of precipitation with animal jelly. In all these he finds the power of the salix to stand very high, only inferior to the tormentil. In some instances we think the author has attempted too general inferences from the result of his experiments, which, however, it is but justice to observe, appear to be conducted with propriety and great impartiality.

That

That the willow bark, properly administered, will cure intermittents, will have a salutary effect in supporting the strength of the constitution under copious suppurations and other debilitating circumstances, is what we do not in the least doubt; but the question more particularly agitated by the author is, the *preference* which he claims for it over the cinchona. On perusing the cases which he gives, some will be found quite unsatisfactory, from the irregularity of the patients; and in those in which the willow bark was of obvious utility, we must confess, that it appears to us that the cinchona, *if genuine and good*, would have produced the same benefit in much less time, provided the patient would have taken it regularly. We must therefore acknowledge the fairness of his cases, at the expence of the inferences which he would deduce from them. As the salix possesses scarcely any sensible *bitterness*, the author evidently is led to undervalue the efficacy of this principle, and yet with some little inconsistency, but (we believe) with practical propriety, he occasionally recommends the addition of quassia to the willow bark, as increasing its virtues, and making it strongly resemble the cinchona.

The great price of this excellent foreign drug, and the extensive and shameful adulterations to which it is often liable, render it highly desirable to increase the number of its substitutes, and to ransack all our fields and woods for indigenous vegetables which may be possessed of similar properties. Perhaps though no single vegetable may unite in itself the powers of the cinchona, a judicious combination may be found to answer the purpose, and the salix certainly deserves a place in our materia medica. Mr. W. exhibits it entirely in the form of decoction, for which the following is his formula.

"℞. Corticis salicis latifoliæ siccati ℥j ß in pulverem crassum redige, et macera in aquæ fontanæ libris duabus per horas sex; deinde coque leni igne per quartam vel tertiam partem horæ, et cola pro usu. Capiat æger cochlearia duo vel tria larga decocti ter vel quater de die; sed febre intermitente, dare oportet unciam unam aut duas secundâ vel tertiâ quaque horâ absente paroxysmo."

---

*Practical Observations on the Management of Ruptures, by* William Hall Timbrel, Esq. *to which are prefixed Two recommendatory Letters, by* William Blair, A. M. Member of the College of Surgeons, &c. &c. Octavo, pp. 94. London, 1803.

This little work is published under the sanction of Mr. Blair, and the approbation of the Society for the Encouragement of Arts, Manufactures, and Commerce, which has honoured the author with their gold medal.

The following directions are given for reducing a rupture:

"In cases of strangulated intestine, or of stricture, the patient should lay on the side of his body *contrary* to that on which the rupture is; by which position, there must be a *lateral* recession of pressure from the aperture, which will give ease when the intestine or omentum cannot from inflammation *return* through the aperture.

"Another position, in cases of difficulty, is to lay upon a chair with its back on the floor, the patient's heels to be placed against the wall, and his head on the ground.

"Let the breath be held in, before an attempt is made to reduce the bowel; for the acts of breathing and speaking, contribute to force down a rupture.

"Cover the fingers with your shirt or handkerchief, by which means the rupture is gathered up with more certainty and dispatch.

"To render the practice easy to every one, I use the expression *knead* the bowel upward through the aperture, as dough is *kneaded*;* but during a state of inflammation, press upon the intestines very gently, if at all.

"By comparing the ruptured side of the body with the sound side, it may be seen and felt when the rupture is reduced.

"Method makes every thing easy, therefore observe the following directions in the *order* in which they are placed:

"1. Lay down. The head is to be lowered, and the knees to be drawn up, or the heels to be raised.

"2. Hold in the breath.

"3. Be perfectly silent.

"4. Cover your fingers with the shirt or handkerchief.

"5. *Knead* up the rupture.

"6. Put on the cushion and truss.

"7. Draw the thigh strap under your thigh *very tight*, and buckle it to the pad."

The author lays the greatest stress on the immobility of the cushion, which he recommends to be made of callico.

---

### MONTHLY REPORTS.

*Cases admitted under the Care of the Surgeon of the Finsbury Dispensary, St. John's Square, Clerkenwell, from August* 10, *to September* 10, 1803.

| | | | |
|---|---|---|---|
| Phlegmone | 1 | Fractura Ulnæ | 1 |
| Abscessus Artuum | 3 | Vulnus | 1 |
| ——— Maxilla | 1 | Luxatio Humeri | 1 |
| Mastodynia | 4 | Lues | 3 |
| Hernia Humoralis | 2 | Gonorrhœa | 2 |
| Ulcera Cruris | 11 | Scrophula | 3 |
| Leucoma | 1 | Ascites | 2 |
| Contusiones | 9 | Hydarthus Genu | 2 |
| | | Sarcocele | |

---

* In the act of kneading, the fingers are to be extended and drawn forwards, gently and shortly.

